# COVID-SCORE: A global survey to assess public perceptions of government responses to COVID-19 (COVID-SCORE-10)

**DOI:** 10.1371/journal.pone.0240011

**Published:** 2020-10-06

**Authors:** Jeffrey V. Lazarus, Scott Ratzan, Adam Palayew, Francesco C. Billari, Agnes Binagwaho, Spencer Kimball, Heidi J. Larson, Alessia Melegaro, Kenneth Rabin, Trenton M. White, Ayman El-Mohandes

**Affiliations:** 1 Barcelona Institute for Global Health (ISGlobal), Hospital Clínic, University of Barcelona, Barcelona, Spain; 2 Graduate School of Public Health & Health Policy, City University of New York (CUNY), New York, New York, United States of America; 3 Department of Social and Political Science, Bocconi University, Milan, Italy; 4 University of Global Health Equity, Kigali, Rwanda; 5 Emerson College, Boston, Massachusetts, United States of America; 6 London School of Hygiene and Tropical Medicine, London, United Kingdom; 7 Department of Health Metrics Sciences, University of Washington, Seattle, Washington, United States of America; Tulane University, UNITED STATES

## Abstract

**Background:**

Understanding public perceptions of government responses to COVID-19 may foster improved public cooperation. Trust in government and population risk of exposure may influence public perception of the response. Other population-level characteristics, such as country socio-economic development, COVID-19 morbidity and mortality, and degree of democratic government, may influence perception.

**Methods and findings:**

We developed a novel ten-item instrument that asks respondents to rate key aspects of their government’s response to the pandemic (COVID-SCORE). We examined whether the results varied by gender, age group, education level, and monthly income. We also examined the internal and external validity of the index using appropriate predefined variables. To test for dimensionality of the results, we used a principal component analysis (PCA) for the ten survey items. We found that Cronbach’s alpha was 0.92 and that the first component of the PCA explained 60% of variance with the remaining factors having eigenvalues below 1, strongly indicating that the tool is both reliable and unidimensional. Based on responses from 13,426 people randomly selected from the general population in 19 countries, the mean national scores ranged from 35.76 (Ecuador) to 80.48 (China) out of a maximum of 100 points. Heterogeneity in responses was observed across age, gender, education and income with the greatest amount of heterogeneity observed between countries. National scores correlated with respondents’ reported levels of trust in government and with country-level COVID-19 mortality rates.

**Conclusions:**

The COVID-SCORE survey instrument demonstrated satisfactory validity. It may help governments more effectively engage constituents in current and future efforts to control COVID-19. Additional country-specific assessment should be undertaken to measure trends over time and the public perceptions of key aspects of government responses in other countries.

## Introduction

COVID-19, the disease caused by severe acute respiratory syndrome coronavirus 2 (SARS-CoV-2), has devastated communities, societies and economies worldwide since its emergence in Wuhan, China, in December 2019. As of 30 June 2020, more than 10 million cases of COVID-19 had been reported in 188 countries and territories, and more than 505,000 deaths had been attributed to the disease [1].

Governments have sought to contain the COVID-19 pandemic by imposing restrictions on activities that enable SARS-CoV-2 to spread rapidly through large networks of people. Common measures have included travel restrictions, closure of schools and places of worship, and stay-at-home orders, although approaches and timetables have differed greatly [2]. Public officials also have promoted the uptake of preventive behaviours such as wearing masks, handwashing, and physical distancing. However, responses to these efforts have varied across settings and age groups [3, 4]. Possible factors for limited or non-compliance include distrust of government [3, 5] and confusion among some segments of the population about conflicting or unclear COVID-19 information received from government sources [6–9].

The degree of public compliance with government orders and prevention recommendations can greatly affect the course of the pandemic, especially as COVID-19 vaccine research is still in its early stages and no definitive treatments are available. The relationship between people’s willingness to comply and their perceptions of how credibly and effectively their governments are responding to COVID-19 warrants careful investigation. Trust in government has been correlated with willingness to adopt protective behaviours in the face of other health threats such as the 2009 H1N1 pandemic [10, 11] and the 2014–2016 West African Ebola epidemic [12]. In the rapidly emerging body of COVID-19 research, a cross-sectional study on Australians’ perceptions of COVID-19 found that individuals who reported higher trust in the government and authorities were more likely to comply with recommended hygienic practices and to avoid physical interaction by cancelling travel plans and staying away from crowds [5].

As this line of research continues to develop, validated instruments will be needed to measure perceptions of government responses to COVID-19 in relation to key domains of performance such as health communication, social welfare and access to healthcare services. A single general tool that can capture this information across multiple countries while being sensitive to language and cultural differences would be of benefit to researchers and policymakers alike. To that end, this paper seeks to validate the COVID-SCORE instrument and report findings on public perception in 19 countries heavily affected by COVID-19.

## Methods

### Ethics statement

This study was approved by Emerson College, USA (IRB protocol number 20-023-F-E6/12) with an expiration date of 11 June 2021. The online questionnaire was administered by Emerson College to gather information from respondents after obtaining their written, informed consent about the survey and this project. Equitable compensation per survey was applied ($2 per complete for Mturk data and increased up to US $3 in some countries) regardless of country being polled to comply with ethical compensation standards. No personally identifiable information was collected or stored.

### Survey instrument design

The instrument was modified from a longer COVID-SCORE-20 survey [13] by an expert panel, following a comprehensive literature review regarding governmental responses to pandemics and other natural disasters. The specific items, although guided by the literature, are original to this study. Each participant responded to a total of 22 items, including the ten COVID-SCORE-10 items and demographic questions, with the US having an additional question (23) on race/ethnicity. The information on age and income was collected through open-ended responses and later coded. Prior to data collection, a pilot study was conducted to test the validity of the content and reliability of the questions with both experts and members of the general public. Data from this pilot study were used to revise the questions in terms of semantics and comprehension.

The survey was presented in American English and Spanish to respondents in the United States, and in British English in the United Kingdom, South Africa, and Singapore. It was presented in British English and Hindi in India, British English and French in Canada, and British English and Hausa in Nigeria. It was presented in the national languages of Brazil, China (Mandarin), Ecuador, France, Germany, Italy, Mexico, Poland, Russia, South Korea, Spain and Sweden. All translators and proofreaders were native speakers who lived in the country to be surveyed. Translated surveys are available in supplementary files. The 19 countries were selected because they were amongst the 25 countries with the highest reported numbers of COVID-19 cases at the time the study was launched (12 June 2020) or, to ensure geographical representation, had the most cases in a WHO region but were not among the countries with the highest number of reported cases worldwide.

### Data collection instrument

COVID-SCORE-10 includes ten items. Each item was selected to assess public perceptions of a key responsibility of government during the pandemic. Responses to each item ranged from “completely disagree” for a minimum score of 1 to “completely agree” for a maximum score of 5. To calculate an overall COVID-SCORE, we summed the responses from each of the ten items and then applied a min-max transformation [14] and multiplied the values by 100 to obtain a score that ranged from 0 to 100. We then calculated the mean score and the associated standard deviation for each country to produce a country-level COVID score.

The COVID-SCORE-10 questionnaire items are:

1. The government helped me and my family meet our daily needs during the COVID-19 epidemic in terms of income, food, and shelter.
2. The government communicated clearly to ensure that everyone had the information they needed to protect themselves and others from COVID-19, regardless of socioeconomic level, migrant status, ethnicity or language.
3. I trusted the government's reports on the spread of the epidemic and the statistics on the number of COVID-19 cases and deaths.
4. The government had a strong pandemic preparedness team that included public health and medical experts to manage our national response to the COVID-19 epidemic.
5. The government provided everyone with access to free, reliable COVID-19 testing if they had symptoms.
6. The government made sure we always had full access to the healthcare services we needed during the epidemic.
7. The government provided special protections to vulnerable groups at higher risk such as the elderly, the poor, migrants, prisoners and the homeless during the COVID-19 epidemic.
8. The government made sure that healthcare workers had the personal protective equipment they needed to protect them from COVID-19 at all times.
9. The government provided mental health services to help people suffering from loneliness, depression and anxiety caused by the COVID-19 epidemic.
10. The government cooperated with other countries and international partners such as the World Health Organization (WHO) to fight the COVID-19 pandemic.

### Random stratified sampling

Strata were established by age (using the following age groups: 18–24, 25–54, 55–64 and 65 years and older); gender (male, female, transgender, and “other,”); and level of education (based on each country’s educational system), which was calculated from data provided by UNESCO, the Organisation for Economic Co-operation and Development, and country data from Sweden, the United Kingdom, and the United States. Educational level was coded into three groups of low, medium and high. “Low” included people who reported not finishing a secondary education (high school); “medium” included those who had completed secondary, vocational, technical, professional associate or high school degree; the “high” group consisted of those who had completed a tertiary or bachelor’s degree and postgraduate work. Each country was divided into regions based on city/town, province or state unit of analysis. The number of participants who could enrol in each of these strata was calculated to reflect the distribution in the general population based on census/survey estimates provided by the World Bank and CIA World Factbook. Data were weighted by strata with each stratum requiring a minimum of 50 participants. These parameters and sources are available in S1 Appendix: Parameters, Sources and External Variables. For the US, race/ethnicity was included in the weighting based on 2018 US Census American Community Survey data (Hispanic, white, black or African American, Asian or Asian Indian, mixed, or “other”) [15].

### Study participants

Participants were recruited through multiple international online panel providers for each country to avoid coverage bias: Dynata provided 6,891 respondents from 19 countries; Opinion Access provided 3,391 respondents from 14 countries; Survey Monkey provided 2,239 responses from 12 countries, and Amazon MTurk provided 905 respondents from 8 large countries to avoid demographic skew. Respondents’ identities were verified using IP addresses and their mobile phones were verified to ensure that each participant was real and unique upon initial registration. Participants were recruited for the panels via a variety of methods, including online, telephone and direct mail solicitation, and equtiably compensated regardless of country being polled in order to comply with ethical compensation standards.

### Data collection

Survey data were collected from 16 to 20 June 2020 from an online panel of 13,426 respondents aged 18 years and older from 19 different countries, ranging between 619 and 773 participants per country. Sample sizes were based on country studies comparing results by demographic characteristic groups (eg age and income groups). The margins of error ranged between 3.5 and 3.9 percentage points. The study utilized a minimum of two online panel providers per country to reduce the coverage bias produced by the proprietary nature of each online panel (except in Sweden where all data were collected by Dynata). In order to reduce the impact of the question order effect, i.e. to limit potential bias influencing answer choices, the order of the ten items of the COVID-SCORE-10, was randomized for each respondent.

### Description of variables

This study employed eight country-specific external variables to further validate the country scores (see S1 Appendix). The three external COVID-19-related variables were:

- Total SARS-CoV-2 positive cases per million persons as reported by Worldometer on 18 June 2020. Worldometer collects COVID-19 data daily from over 5,000 sources, including ministries of health, other government institutions, and local media sources in real-time, and the website’s data have been cited in more than 6,000 peer-reviewed journal articles [16, 17].
- Total SARS-CoV-2 deaths per million persons as reported by Worldometer on 18 June 2020 [16, 17].
- The proportion of “yes” responses per country to a study instrument item asking whether the respondent or a family member had been sick with COVID-19, measured as “yes”, “no”, and “unsure.”

For further external validation of country scores, three variables were included to measure the socioeconomic and democratic performance of each country:

- The World Bank country income level was chosen as it provides an indication of the country’s overall economic performance relative to all other national economies. The World Bank classifies national economies based on their gross national income per capita, converted to US$ for global comparison using the Atlas method [18], according to thresholds adjusted for annual inflation representing high, upper-middle, lower-middle, and low-income economies [19].
- The 2019 Economist Intelligence Unit (EIU) Democracy Index score for each country, which is measured on a continuous scale from 0 to 10, was included to assess how more and less democratic societies evaluate their government’s COVID-19 response. This index’s values correspond to four types of regimes: full democracies (scores greater than 8), flawed democracies (scores greater than 6 and less than or equal to 8), hybrid regimes (scores greater than 4, and less than or equal to 6), and authoritarian regimes (scores less than or equal to 4) [20].
- The country’s Human Development Index (HDI) score for 2019 from the United Nations Development Programme, which is measured on a continuous scale of 0–1. The HDI is a composite index that combines health (life expectancy at birth), education (expected and mean years of schooling) and economic performance (per capita gross national income) indicators to measure a country’s average human development [21].

Two additional variables were included to estimate the level of public trust in the government:

- The proportion of those surveyed in each country who responded having either “a lot” or “some” trust in the national government (as opposed to “not much” and “not at all”) reported in the Wellcome Global Monitor 2018 report, which was used for external validation of COVID-SCORE-10. This global annual survey measures public perceptions of key health system actors, including governments, regarding science and health challenges. Responses from China were not available, and China was therefore excluded from analyses using this variable [22].
- The proportion of those surveyed responding “yes” to an item in the study instrument that assesses trust in the government to successfully address unexpected health threats, including COVID-19,which was used in this study to internally validate COVID-SCORE-10.

### Analysis plan

We evaluated the distribution of the country score and the items that constitute the score descriptively by country. We then examined whether the results varied by gender, age group, education level, and monthly income above/below the median. We also examined the internal and external validity of the index using the appropriate predefined variables.

To test the internal validity of COVID-SCORE-10, we measured Cronbach’s alpha for the ten items that comprised the final score. To test for dimensionality of the results, we used a principal factor analysis (PCA) for the ten survey items. Through univariate regression and correlation analyses, we also considered the association of the score with the country-level proportion of people responding “yes” to having trust in their government to successfully address unexpected health threats, including COVID-19.

To test the external validity, we considered the association between the score and predefined variables to evaluate how the score varied with established indicators and associative questions. The independent variables that were used for external validation were the World Bank income classification level, the EIU Democracy Index score, the HDI score, country mean trust in the national government as measured by responses to the Wellcome Global Monitor score, the proportion of individuals who report themselves and/or their family members as having been sick with COVID-19, cases per million from Worldometer.com, and mortality per million from Worldometer.com.

We fit a univariate regression model for each of these variables with the country COVID-SCORE-10 as the dependent variable and report the beta coefficient with 95% confidence intervals. We also present the Pearson correlation coefficients for all validating variables that are continuous. All data used for the associations with the external and internal validation are measured at the country level as we sought to validate the instrument for measurement at a country level.

## Results

### Average COVID-SCORE by country and item

Overall, average country-level scores (SD) ranged from 35.76 (SD = 23.05) points to 80.48 (SD = 16.31) points out of 100, with higher scores indicating that, on average, respondents across the country perceived their government’s response to COVID-19 as more adequate (Table 1). China had the highest score, 80.48 (SD = 16.31), followed by South Korea with 74.54 (SD = 18.61) and South Africa with 64.62 (SD = 22.94). The three lowest-scoring countries were Ecuador with a score of 35.76 (SD = 0.68), Brazil with 36.35 (SD = 024.59) and Poland with 41.28 (SD = 25.30). Higher-scoring countries were primarily located in Asia while lower-scoring countries were found in Latin America and Europe.

**Table 1:** Average score (standard deviation) by country (n = 19) for each COVID-SCORE (n = 10) item.

Items Country	1. The Government helped me and my family meet our daily needs during the COVID-19 epidemic in terms of income, food, and shelter.	2. The government communicated clearly to ensure that everyone had the information they needed to protect themselves and others from COVID-19, regardless of socioeconomic level, migrant status, ethnicity or language.	3. I trusted the government's reports on the spread of the epidemic and the statistics on the number of COVID-19 cases and deaths.	4. The government had a strong pandemic preparedness team that included public health and medical experts to manage our national response to COVID-19 epidemic.	5. The government provided everyone with access to free, reliable COVID-19 testing if they had symptoms.	6. The government made sure we always had full access to the healthcare services we needed during the epidemic.	7. The government provided special protections to vulnerable groups at higher risk such as the elderly, the poor, migrants, prisoners and the homeless during the COVID-19 epidemic.	8. The government made sure that healthcare workers had the personal protective equipment they needed to protect them from COVID-19 at all times.	9. The government provided mental health services to help people suffering from loneliness, depression and anxiety caused by the COVID-19 epidemic.	10. The government cooperated with other countries and international partners such as the World Health Organization (WHO) to fight the COVID-19 pandemic.	Mean COVID-SCORE (SD)
Brazil (n = 717)	2.64 (1.30)	2.68 (1.45)	2.53 (1.31)	2.50 (1.35)	2.15 (1.29)	2.52 (1.32)	2.43 (1.31)	2.61 (1.35)	2.11 (1.21)	2.38 (1.31)	36.35 (24.59)
Canada (n = 707)	3.43 (1.22)	3.68 (1.36)	3.62 (1.21)	3.49 (1.22)	3.47 (1.26)	3.55 (1.21)	3.12 (1.22)	3.26 (1.22)	3.10 (1.11)	3.67 (1.07)	61.00 (21.88)
China (n = 712)	4.04 (0.97)	4.28 (0.90)	4.25 (0.89)	4.37 (0.85)	4.29 (0.90)	4.26 (0.87)	4.05 (0.93)	4.23 (0.89)	4.00 (0.95)	4.40 (0.77)	80.48 (16.31)
Ecuador (n = 741)	2.08 (1.19)	2.90 (1.36)	2.17 (1.24)	2.18 (1.26)	2.27 (1.37)	2.64 (1.37)	2.59 (1.32)	2.31 (1.32)	2.32 (1.27)	2.85 (1.28)	35.76 (23.05)
France (n = 669)	2.76 (1.26)	3.06 (1.29)	3.03 (1.32)	3.10 (1.24)	2.89 (1.29)	3.20 (1.25)	2.96 (1.26)	2.42 (1.29)	2.79 (1.06)	3.48 (1.08)	49.20 (22.07)
Germany (n = 722)	3.19 (1.33)	3.70 (1.19)	3.70 (1.30)	3.64 (1.21)	3.24 (1.35)	3.64 (1.24)	3.32 (1.22)	3.22 (1.25)	2.93 (1.20)	3.95 (1.09)	61.32 (22.20)
India (n = 742)	3.38 (1.27)	3.65 (1.22)	3.61 (1.26)	3.59 (1.21)	3.43 (1.30)	3.59 (1.22)	3.49 (1.29)	3.64 (1.21)	3.33 (1.28)	3.84 (1.78)	63.88 (24.07)
Italy (n = 736)	2.89 (1.22)	3.36 (1.21)	3.43 (1.17)	3.35 (1.15)	2.75 (1.30)	3.02 (1.22)	2.71 (1.18)	2.89 (1.24)	2.71 (1.19)	3.58 (1.08)	51.71 (21.25)
Mexico (n = 699)	2.24 (1.32)	3.28 (1.45)	2.90 (1.45)	3.12 (1.40)	2.31 (1.41)	3.06 (1.44)	2.81 (1.45)	2.71 (1.45)	2.75 (1.39)	3.41 (1.30)	46.48 (26.84)
Nigeria (n = 670)	1.97 (1.30)	3.78 (1.30)	2.77 (1.37)	2.90 (1.41)	2.87 (1.53)	2.66 (1.45)	2.44 (1.40)	3.04 (1.41)	2.31 (1.37)	3.80 (1.24)	46.32 (22.71)
Poland (n = 666)	2.67 (1.28)	3.03 (1.37)	2.41 (1.38)	2.86 (1.31)	2.48 (1.37)	2.37 (1.36)	2.61 (1.41)	2.46 (1.37)	2.28 (1.25)	3.32 (1.21)	41.28 (25.30)
Russia (n = 680)	2.65 (1.27)	3.48 (1.25)	2.95 (1.32)	3.08 (1.32)	2.70 (1.33)	2.63 (1.33)	2.90 (1.35)	2.94 (1.32)	2.60 (1.18)	3.64 (1.04)	48.85 (24.03)
Singapore (n = 752)	2.68 (1.33)	3.91 (1.23)	3.31 (1.35)	3.46 (1.28)	3.14 (1.44)	3.30 (1.31)	3.16 (1.31)	3.27 (1.31)	2.64 (1.31)	4.14 (1.00)	57.55 (21.76)
South Africa (n = 655)	3.49 (1.37)	3.63 (1.14)	3.85 (1.16)	3.93 (1.04)	3.75 (1.14)	3.71 (1.15)	3.40 (1.11)	3.39 (1.21)	3.07 (1.13)	3.63 (1.11)	64.62 (22.94)
South Korea (n = 619)	3.67 (1.02)	3.98 (1.01)	4.01 (0.99)	3.93 (1.05)	3.93 (1.07)	4.19 (0.87)	3.94 (1.01)	4.34 (0.82)	3.70 (1.05)	4.12 (0.87)	74.54 (18.61)
Spain (n = 748)	2.63 (1.33)	3.11 (1.48)	2.83 (1.44)	3.01 (1.49)	2.09 (1.33)	3.06 (1.41)	2.65 (1.39)	2.41 (1.35)	2.61 (1.33)	3.46 (1.27)	44.68 (25.91)
Sweden (n = 650)	2.56 (1.21)	3.10 (1.31)	3.42 (1.41)	2.86 (1.32)	2.11 (1.28)	2.65 (1.34)	2.30 (1.21)	2.30 (1.24)	2.37 (1.44)	3.14 (1.23)	42.07 (23.14)
UK (n = 768)	3.04 (1.24)	3.17 (1.30)	3.04 (1.33)	2.83 (1.32)	2.63 (1.36)	3.09 (1.30)	3.00 (1.29)	2.65 (1.35)	2.70 (1.20)	3.31 (1.19)	48.66 (24.28)
US (n = 773)	3.16 (1.34)	3.14 (1.35)	3.10 (1.33)	3.05 (1.40)	2.92 (1.45)	3.01 (1.39)	2.97 (1.41)	3.03 (1.44)	2.82 (1.40)	3.03 (1.39)	50.57 (28.99)
Average score per item and overall COVID-SCORE (SD)	2.91 (1.35)	3.41 (1.33)	3.20 (1.39)	3.22 (1.36)	2.91 (1.45)	3.16 (1.38)	2.99 (1.36)	3.00 (1.40)	2.79 (1.31)	3.53 (1.25)	52.95 (26.20)

Of the ten individual COVID-SCORE items (see Table 1), the highest-scoring item across all countries was item 10 (Perceived multilateral (WHO) and international cooperation) with an average score of 3.53 (1.24) on the scale of 1 to 5. The lowest-scoring item was item 9 (addressing mental health service availability during the pandemic) with an average score across all countries of 2.79 (1.31).

### Demographic factors

**Fig 1:**
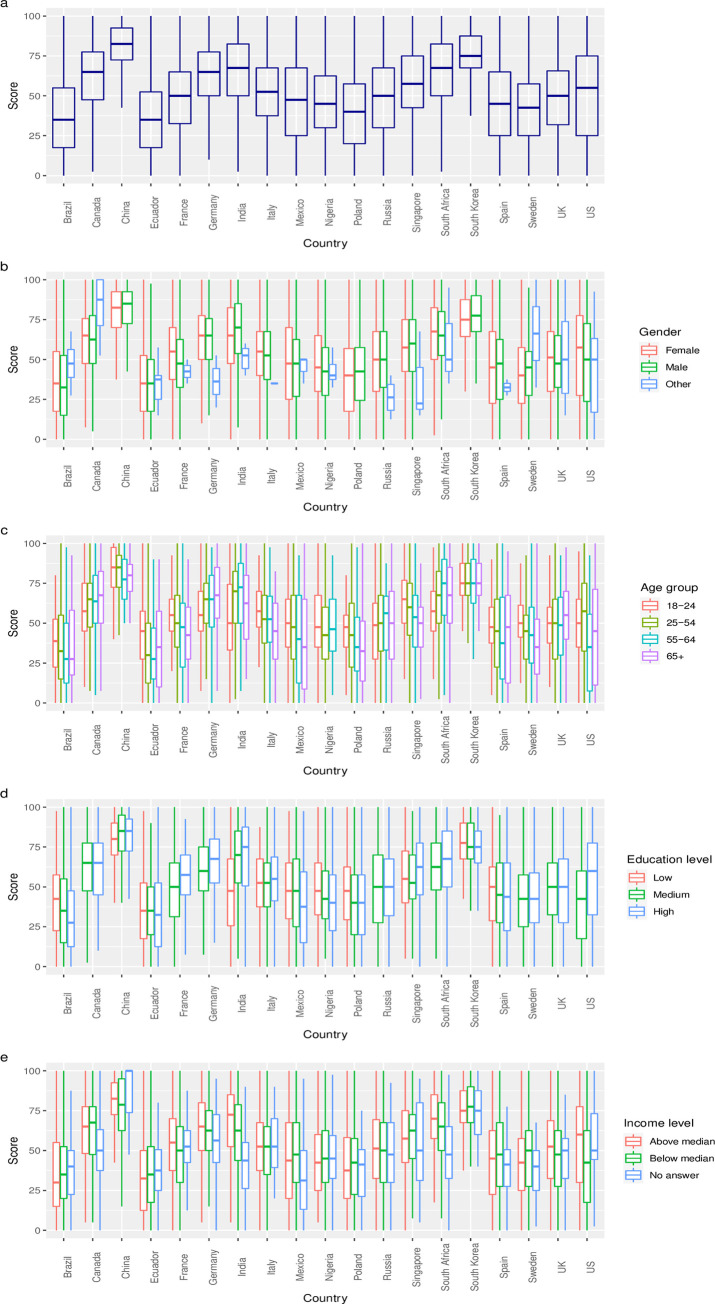
Box and whiskers plot of country score and stratification of demographic variables of interest.

Although there were important differences in the distribution of COVID-SCORE values across countries (Fig 1A), scores did not vary significantly across gender, age group, education, or income level, as illustrated in the boxplots in Fig 1B–1E respectively.

Fig 1A summarizes the distribution of individual responses to COVID-SCORE that make up the group means presented in Table 1. Greater heterogeneity among responses was observed in seven countries with wider boxplots (Brazil, Ecuador, Mexico, Poland, Sweden, the UK, and the US). The country with the most variability in its responses was the US as there is a uniform distribution of people rating the government response well and a similar distribution scoring it poorly. Compared to this apparent lack of population consensus about the quality of government response, the lowest amount of variability in scores was seen in China and South Korea, indicating greater homogeneity in perceptions among responses, which tended to be very positive. The scores for the stratifying variables (i.e. age, gender, education and income levels) are tightly grouped (Fig 1B–1E) with some within-country variation for education and income. For example, in India those with middle and high education levels scored the government higher, whereas those with a low education level had greater variability in their scores, and in the United States those earning above the monthly median income scored the government higher than those earning below.

The line through each box is the median, the limits of the box are the interquartile range, and the whiskers on each box show the 5^th^ and 95^th^ percentile of the data. Panel a is the distribution of all responses for each country. Panel b stratifies the scores by gender, Panel c by age group, Panel d by education level, and Panel e by the respondents’ variance from the national median income.

### Validation of the score

Examining the internal validity of the score, we found that Cronbach’s alpha was 0.92 and that the first component of the PCA explained 60% of variance with the remaining factors having eigenvalues below 1, strongly indicating that the tool is both reliable and unidimensional. The PCA found that items 6, 4, and 7, respectively, contributed the most weight to the first component with items 1, 10, and 2, respectively, contributing the least weight. Additional correlations and outputs from simple regressions are presented in Table 2. From the perspective of internal validity, we also found that the proportion of “yes” responses to the survey item assessing “trust in the government to successfully address unexpected health threats, including COVID-19” had a strong correlation with the mean country score (r = 0.64, β = 45.8 [20.0, 71.7]). In assessing external validity, we found that there were moderate and negative associations between the country score and the proportion of participants who reported that “they and/or their family members had been sick with COVID-19” (r = -0.34, β = -51.2 [-120.3, 17.9]) as well as for COVID-19 cases per million (r = -0.37, β = -0.0019 [-0.004, 0.0005]). A weak and negative correlation was found for the mortality from COVID-19 per million (r = -0.16, β = -0.01 (-0.003, 0.02). A moderately strong positive correlation was found for the measure of general trust in the national government (Wellcome) (r = 0.58, β = 37.6 [10.1, 65.1]). The country score did not have any observed correlations with the country HDI score (r = 0.07, β = 7.3 [-43.5, 58.1]) or with the EIU Democracy Index score (r = 0.04, β = 0.26 [-2.7, 3.2]). No relationship was found between World Bank income level and the mean country score.

**Table 2:** Association between external validation variables (n = 8) and the overall country score.

Variable	beta coefficients from univariate regressions (95% CI)	Pearson Correlation coefficient (r)
Population trust in govt to successfully respond to unexpected health threats (survey question)^a^	45.8 (20.0, 71.7)	0.64
Percentage of respondents reporting COVID-19 illness for themselves and/or family	-51.2 (-120.3, 17.9)	-0.34
Country-specific COVID-19 cases per million (Worldometer data)	-0.0019 (-0.004, 0.0005)	-0.37
Country-specific COVID-19 mortality per million (Worldometer data)	-0.01 (-0.03, 0.02)	-0.16
General trust in the national government (Wellcome Global Monitor)	37.6 (10.1, 65.1)	0.58
Human Development Index score 2019	7.3 (-43.5, 58.1)	0.07
EIU Democracy Index score 2019	0.26 (-2.7, 3.2)	0.04
World Bank country income level 2019	Low vs middle 2.0 (-17.4, 21.4)	NA
	middle vs high -1.5 (-14.3, 11.3)	

^a^ The variable “Population trust in govt to successfully respond to unexpected health threats” was collected via the study survey and evaluated as an external validation variable, being external to the COVID-SCORE instrument itself.

## Discussion

Governmental responses to the COVID-19 pandemic have varied widely across countries. We set out to measure public perceptions of government COVID-19 pandemic response efforts in 19 of the most heavily affected countries and to identify the key factors associated with those perceptions. Our goal was to provide governments with a survey instrument that they could use to support the upgrade of measures to improve public commitment to disease prevention and control, and to mitigate the social, health and economic impacts of COVID-19. To that end, we developed and applied a novel instrument, COVID-SCORE-10, to measure public perception of ten key responsibilities of government.

We found that COVID-SCORE-10 is reliable and unidimensional. At the country level, age, gender, education, income, World Bank income level, level of human development, and degree to which a country is democratic were not associated with the overall country score. Mortality per million from COVID-19, the proportion of respondents who reported that they or a family member had become sick with COVID-19, trust in the national government, as measured by the Wellcome Global Monitor, and trust in the government to successfully address unexpected health threats, as measured in this study, were associated with the COVID-SCORE in expected directions.

For example, we found there was a negative correlation between a country’s median score and both its number of COVID-19 cases per million (-0.36) and the proportion of survey respondents and family members who had COVID-19 (-0.34). Conversely, we observed that higher mean country scores were associated with fewer cases per million and a smaller percentage of people who said they or a family member had been affected by COVID-19.

Overall, a higher mean country score was strongly associated with measures of trust. General trust in the national government and trust in the government to successfully address unexpected health threats were clearly associated with country scores for COVID-SCORE-10. This finding is consistent with data from 19 European countries which show in a non-peer-reviewed preprint that, in geographical regions where higher levels of trust in government had been documented prior to the COVID-19 pandemic, there were greater reductions in behaviour such as non-essential local travel during March 2020, perhaps indicative of increased compliance with the lockdowns that European countries were imposing in response to sharp increases in COVID-19 cases at that time [23].

In published studies of previous health emergencies, trust in the credibility of public health experts, health systems and scientific evidence has been shown to encourage appropriate utilization of medical services (e.g. voluntary medical testing), compliance with lockdowns, and adoption of preventive public health measures, such as physical distancing and mask-wearing, all of which are difficult and costly to implement without public support and commitment. However, managing a pandemic successfully requires large-scale behavioural changes individually, organizationally and societally, and these extend beyond handwashing, facemasks, and self-isolation. Moreover, crises, and particularly epidemics, raise real barriers to efforts to align individual and collective interests, which challenge the adoption of behavioural changes that are essential to prevent the spread of disease [24]. In this context, governments bear great responsibility to communicate effectively the collective benefits of adhering to evidence-based measures, and public trust in government is an essential component of this process [25]. Lack of trust during the COVID-19 pandemic has been associated with poorer mental health [26] and has historically undermined the health and health-seeking behaviours of ethnic minorities and others who have experienced discrimination in the health system [27–29].

Within the literature on policy, trust is related to cultural norms, values, and beliefs, and it is regarded as a component of key social and economic policy outcomes [30–32]. These factors may help explain the geographical differences in the country scores reported here. For example, citizens of many Asian countries, including those in this study, trusted more in the government [33]. Countries in Asia initially better contained COVID-19 compared to countries in Europe and the Americas, utilising massive testing campaigns, contact tracing, and aggressive lockdown policies [34]. In contrast, some countries in Latin and North America and Europe substantially delayed imposing any restrictions at all and were heavily criticized for their response to the pandemic [35, 36]. It is noteworthy that the Democracy Index did not correlate with the overall score of populations’ perception of the governmental response, but trust in government did, which appears to be independent of system of governance in that regard [37].

Effective, credible, consistent and culturally informed health communication is vital in influencing positive health behaviours [32, 38, 39], especially in terms of encouraging people to adhere to COVID-19 control measures. Failure by governments to implement targeted public health communication measures can undermine their and the health system’s pandemic responses. During the course of this pandemic, it has too often been difficult for the public to distinguish between evidence-based and less scientifically reliable information, in part due to poor messaging by health authorities and other officials [40, 41] and uncertain and evolving scientific information, including high-profile retractions of research papers [42, 43] and conflicting modelling analyses [44]. Of the 10 items in our study, item 2, which directly measures government communication responsibilities, scored as the second highest-rated item across all countries. Other items indirectly related to communication responsibilities also scored well. The item assessing public perception of government reports and statistics, key components of a responsible government communication strategy, scored above average globally. Participants gave the highest score to the belief that their government was cooperating with WHO and other countries, though it is an item from which they are far removed. Our results indicate that, in these countries, the public perceives that their government is collaborating with international partners, including WHO, and it is important insofar as these organizations play a major role by providing clear, unbiased guidance [45, 46].

Disease control measures are further challenged by the unequal risks and burdens of COVID-19 as well as access to resources to prevent the spread of the disease. For example, families without running water cannot wash hands, the homeless cannot shelter in place, those in prisons and high-density urban slums cannot physically distance, low-wage and essential workers cannot telework, and overcrowding in reception and detention centres may increase exposure of migrants and refugees to the disease [47, 48]. Absence of health insurance or reduced access to healthcare in disadvantaged and marginalized and distrustful communities, even in countries with universal healthcare, may prevent individuals from seeking treatment. Restrictions on public transport to achieve physical distancing may consequently prevent individuals from being able to pick up prescription medications or access necessary health services. The absence of adequate protection measures will continue to disproportionately affect minority groups [48]. Moreover, there is a need to protect people living in long-term health facilities, especially the elderly, and their caregivers, who are also disproportionately affected [49].

As COVID-19 quickly and inexorably spread around the world and the economic crisis deepened, public health services were overwhelmed and health gaps grew within and among countries [50]. Study results of the items comprising COVID-SCORE-10 support the need to strengthen health system functions during the pandemic. Globally, item 6 (assessing continued access to necessary services) was scored (3.16) closest to the mean of all items (3.11) and was scored above 4.00 only in China and Singapore. Other responsibilities related to health systems functions such as item 5 (assessing access to free, reliable COVID-19 testing) (2.91) and item 8 (assessing the provision of personal protective equipment to healthcare workers) (3.00) received among the lowest scores. It is understandable that meeting the mental health needs of the population was scored the lowest (2.79), given the increase in anxiety and depression during the pandemic [51], but it also exposes the underlying weaknesses of mental health services in a number of settings, as well the global challenges of fragmented health systems to reach populations that need mental health services [52].

While lockdown strategies may be effective in reducing the spread of COVID-19 and maintaining general health system responsiveness, this single-disease response compromises care for a range of other health conditions. The effects of health service disruption have already been reported for HIV, TB, and malaria care in high-burden settings [53]. Reinvestment in public health and outreach services is warranted to better prepare for and manage future crises [54] as a wide range of services have been impacted by recent budget cuts in several countries [55].

In our study, the global mean for item 7 (assessing socioeconomic and health protections for vulnerable groups) (2.99), and item 1 (assessing assistance meeting daily needs in terms of income, food, and shelter) (2.90) were scored substantially below the means for all other items. These findings underscore the need to give special attention to vulnerable groups as many countries attempt to reopen their economies, although SARS-CoV-2 continues to spread globally, and the risk of recurrence in countries that have controlled transmission remains.

The apparent reliability of this new instrument suggests that it may be a new tool that governments can refine and use to monitor how individuals and communities perceive their country’s response to COVID-19. The internal validity of the instrument is strongly supported by the value of Cronbach’s alpha for the survey instrument and the results of the PCA indicating that it is unidimensional. The external validity of the score is also supported by the results as the score was correlated with valid external indicators in the expected direction. Our data suggest that both the overall ratings and the degree of variability in responses offer valuable guidance to decisionmakers. These scores are not meant to compare countries to one another, but rather to help governments track and measure changes within their country over time and identify regions or groups that may require additional investment or modified interventions. Longitudinal studies using this instrument at recurring intervals could measure variations in public perceptions over time as a country’s epidemiological trajectory and response strategies change. Such studies would also provide insight to the instrument’s potential for continuous use as a monitoring tool. COVID-SCORE-10 could help governments to improve their health care system’s resilience by identifying potential sources of communication breakdown, strengthening their capacity to respond in future crises, and evaluating the efficacy and potential replicability of pandemic management strategies used to promote physical and mental health system resilience in other countries [56].

The findings of this study are limited due to cross-sectional data, which do not permit causal claims to be made between these scores and external variables. Interpretation of “government responses” must be broadly considered given that governance structures (including subnational governments) vary in their pandemic responsibilities. Furthermore, although we have taken precautions to achieve representative samples from each country, the methodological limitations of inadequate representation of some segments of a society, such as those most marginalized, may have introduced a response bias that could have altered their country’s summative score or its associations with other variables.

## Conclusion

Effective control of COVID-19 requires governments and their constituencies to engage in mutually trusting relationships with a shared understanding of what is expected by both sets of actors. The ability of government and public health leaders to gauge how the population perceives the effectiveness of government responses to COVID-19, both generally and on specific responsibilities, is essential for identifying potential obstacles to achieving disease control objectives. The 10-item instrument that we have validated in this study is easy to administer and yields simple scores that can inform policy debates and can guide the design and implementation of COVID-19 prevention, testing and treatment initiatives including communications initiatives. COVID-SCORE also has a wide range of research applications as political and social scientists continue to explore how trust in government influences public behavior in the context of major health threats. Although the instrument was used for cross-sectional data collection in this study, we see value in applying it longitudinally as both the COVID-19 pandemic and policy responses to the pandemic continue to evolve in different national contexts. Given the importance of how subnational governments are responding to COVID-19 in large countries with multiple geographically distinct pandemic trajectories, further research should also explore how COVID-SCORE can contribute to subnational knowledge generation and policy-making.

## Supporting information

S1 Checklist STROBE checklist. (DOCX)

S1 Appendix Parameters, sources and external variables. This file contains the population parameters for each country used to ensure a stratified sampling of respondents by gender, age, education level, region, and income level. It also contains the country data for each of the external variables considered in the analysis.
(XLSX)

S2 Appendix Respondent characteristics. This file contains the population characteristics stratified by age, education, gender and income (and race in the United States) as well as raw responses to each COVID-SCORE item per country.
(XLSX)

S1 Study questionnaire This file contains the 22 items (23 for United States) that composed the online survey, including the 10 items of COVID-SCORE. (DOCX)
