# Supplementary material for: COVID-SCORE: A global survey to assess public perceptions of government responses to COVID-19 (COVID-SCORE-10)
Source: PLoS One. 2020 Oct 6;15(10):e0240011. doi: 10.1371/journal.pone.0240011 (PMC7538106; doi:10.1371/journal.pone.0240011)
Supplement: S1 Checklist — (DOCX) [file pone.0240011.s001.docx]

STROBE Statement Checklist for

“COVID-SCORE: A global survey to assess public perceptions of government responses to COVID-19”

| *Checklist Item* | *Page no.* | *Description of how addressed in this manuscript* |
| --- | --- | --- |

**Title and abstract**

| Title | 1 | Study design indicated by “survey” in title. |
| --- | --- | --- |
| Abstract | 1 | Abstract provides concise, informative and balanced summary of methods and findings. |

**Introduction**

| Background | 2 | Intro explains the scientific background and rational for measuring public perception of government pandemic responses. |
| --- | --- | --- |
| Objectives | 2 | Study objectives and hypothesis reported in final paragraph of Introduction. |

**Methods**

| Study design | 2-5 | Key elements of study design reported (collection and aggregation of public perception at country level, creation and internal validation of index score, external validation of index score with key population-level measures.) |
| --- | --- | --- |
| Setting | 3-4 | Settings (n=19) and dates of survey (June 16-20, 2020) provided. |
| Participants | 3-4 | Eligibility criteria and random stratified sampling method for participant inclusion reported. |
| Variables | 4-5 | All variables are clearly defined as outcome (final score) or validation variables |
| Data sources | 4-5; S1 Appendix | Sources for each variable provided. |
| Bias | 2-4 | Response and coverage biases were avoided by using the random stratified sampling method, verification of individual respondents, use of multiple data panels where possible, and translation of the instrument to the local language. |
| Study size | 4 | Study size was determined by |
| Quantitative variables | 3-5 | Outcome variable (COVID-SCORE) aggregated to country level and analyzed using external validation variables with groupings for World Bank income classification, above/below median income, education and gender specified. |
| Statistical methods | 5 | The score was calculated through summation and fit to range from 0 to 100 through a min-max transformation. Chronbach’s alpha was calculated to test the internal validity of COVID-SCORE, and its dimensionality was tested using principal component analysis. The arithmetic mean of the score for each country was regressed against all validation variables and correlated with validation variables that were not continuous. We report the beta coefficients from these regressions and the associated 95% confidence interval. |

**Results**

| Participants | 4 | All participant responses were analyzed as the methods chosen for participant selection precluded loss to follow-up or multiple stages of observation. |
| --- | --- | --- |
| Descriptive data | S5 Appendix 2 | Respondent demographic information and number of responses per COVID-SCORE item provided. |
| Outcome data |  | The outcome variable is the COVID-SCORE in a country, as calculated through summation, min-max transformation, and mean across all respondents in that country. |
| Main results | 3-4; S2 Table | Arithmetic mean of COVID-SCORE for each country and the associated standard deviation. |
| Other analyses | N/A | N/A |

**Discussion**

| Key results | 9 | Key results were the mean of the country scores and the instrument validation and correlation in expected direction for external variables. |
| --- | --- | --- |
| Limitations | 9 | Cross-sectional data prohibits any causal interpretation of the results, and sampling method does not ensure inclusion of segments of the population most at risk for COVID-19 i.e. random sampling does not eliminate the possibility of some selection bias. |
| Interpretation | 6-9 | Discussion cautiously interprets the results of the analyses in connection with the literature. |
| Generalisability | 9 | This survey was conducted at a single point during the pandemic and while generalisable, it will be more so through continued use of the instrument with longitudinal analyses. |

**Other information**

| Funding | 13 | Funding for this international study was made possible by unrestricted grants from the City University of New York (CUNY) Foundation, www.rfcuny.org/RFWebsite. CUNY (SR and AE) received three grants to make its contribution possible including: US $25,000 from Dr. Jonathan Fielding, $10,000 from the United States Council for International Business Foundation, www.uscib.org, and $4,000 from Dr. Kenneth Rabin. The CUNY Foundation contributed $25,000 from budget account number 90057-00-99. Bocconi University, Italy, www.unibocconi.eu, (FCB and AM) contributed EUR 10,000 to the study. Emerson College, USA (SK) was the funding recipient. The authors are solely responsible for all content, and non-authoring funders played no role in study design, data collection and analysis, decision to publish, or preparation of the manuscript. |
| --- | --- | --- |
