# Supplementary material for: COVID-SCORE: A global survey to assess public perceptions of government responses to COVID-19 (COVID-SCORE-10)
Source: PLoS One. 2020 Oct 6;15(10):e0240011. doi: 10.1371/journal.pone.0240011 (PMC7538106; doi:10.1371/journal.pone.0240011)
Supplement: S1 Study questionnaire — (DOCX) [file pone.0240011.s004.docx]

**General public version of COVID-SCORE**

The purpose of the survey is to measure resident’s reactions to their government’s COVID-19 response efforts. The risks in this routine survey are considered minimal. You may feel uncomfortable considering the implications of COVID-19 and conveying information about themselves and their Government. Participation in this research study is completely voluntary and you may discontinue participation at any time, and refusal to participate will involve no penalty or loss of benefits to which the subject is otherwise entitled. Your responses will remain anonymous and confidential. The survey should take less than 4 minutes to complete. By answering the questions you are indicating that you have read the description of the study, are over the age of 18, and that you agree to the terms as described.

If you have questions about your rights as a research participant, you may contact the Emerson College Institutional Review Board (IRB), which is concerned with the protection of volunteers in research projects. You may reach the lead investigator via email at emersonpolling@emerson.edu or the Chair of the IRB by emailing human_subjects@emerson.edu.

Thank you in advance for your participation!

1. To start, do you trust that your government will successfully address unexpected health threats to our nation, including COVID 19 epidemic?

Yes

No

2. Have you or anyone in your family gotten sick with COVID-19 since the start of the pandemic?

Yes

No

Unsure

*(randomize 3-12)*

3. Now, on a scale of 1 to 5, how would you rate your government’s response to COVID-19-epidemic. To begin, the Government helped me and my family meet our daily needs during the COVID-19 epidemic in terms of income, food, and shelter.

Completely disagree

Somewhat disagree

Neutral/no opinion

Somewhat agree

Completely agree

4. The government communicated clearly to ensure that everyone had the information they needed to protect themselves and others from COVID-19, regardless of socioeconomic level, migrant status, ethnicity or language.

Completely disagree

Somewhat disagree

Neutral/no opinion

Somewhat agree

Completely agree

5. I trusted the government’s reports on the spread of the epidemic and the statistics on the number of COVID-19 cases and deaths.

Completely disagree

Somewhat disagree

Neutral/no opinion

Somewhat agree

Completely agree

6. The government had a strong pandemic preparedness team that included public health and medical experts to manage our national response to COVID-19 epidemic.

Completely disagree

Somewhat disagree

Neutral/no opinion

Somewhat agree

Completely agree

7. The government provided everyone with access to free, reliable COVID-19 testing if they had symptoms.

Completely disagree

Somewhat disagree

Neutral/no opinion

Somewhat agree

Completely agree

8. The government made sure we always had full access to the healthcare services we needed during the epidemic.

Completely disagree

Somewhat disagree

Neutral/no opinion

Somewhat agree

Completely agree

9. The government provided special protections to vulnerable groups at higher risk such as the elderly, the poor, migrants, prisoners and the homeless during the COVID-19 epidemic.

Completely disagree

Somewhat disagree

Neutral/no opinion

Somewhat agree

Completely agree

10. The government made sure that healthcare workers had the personal protective equipment they needed to protect them from COVID-19 at all times.

Completely disagree

Somewhat disagree

Neutral/no opinion

Somewhat agree

Completely agree

11. The government provided mental health services to help people suffering from loneliness, depression and anxiety caused by the COVID-19 epidemic.

Completely disagree

Somewhat disagree

Neutral/no opinion

Somewhat agree

Completely agree

12. The government cooperated with other countries and international partners such as the World Health Organization (WHO) to fight the COVID-19 pandemic.

Completely disagree

Somewhat disagree

Neutral/no opinion

Somewhat agree

Completely agree

13. Now on the same scale of 1 to 5, how would you rate the business response to COVID-19.

Do you believe that the Government and business community in your country are working together to restart the economy safely after COVID-19.

Completely disagree

Somewhat disagree

Neutral/no opinion

Somewhat agree

Completely agree

14. Information from businesses in my country about the economic recovery from COVID-19 is reliable.

Completely disagree

Somewhat disagree

Neutral/no opinion

Somewhat agree

Completely agree

15. I trust my employer to determine the necessary requirements to protect me, my colleagues, and our customers at our workplace.

Completely disagree

Somewhat disagree

Neutral/no opinion

Somewhat agree

Completely agree

16. I would follow my employer’s recommendation to get a COVID-19 vaccine once the government has approved it as safe and effective.

Completely disagree

Somewhat disagree

Neutral/no opinion

Somewhat agree

Completely agree

17. If a COVID-19 vaccine is proven safe and effective and is available to me, I will take it.

Completely disagree

Somewhat disagree

Neutral/no opinion

Somewhat agree

Completely agree

18. What is your age? ____

19. What is the highest degree or grade level you completed? (Country Specific)

20. What is your gender?

Female

Male

Transgendered

Other

21. What is your average monthly household income?_______________________

22. What region do you live in? *(Country Specific)*

23. For statistical purposes only, can you please tell me your ethnicity? *(US Only)*

Hispanic or Latino of any race

White or Caucasian

Black or African American

Asian

Other or multiple races
